# The Impact of Mesothelin in the Ovarian Cancer Tumor Microenvironment

**DOI:** 10.3390/cancers10090277

**Published:** 2018-08-21

**Authors:** Tyvette S. Hilliard

**Affiliations:** Department of Chemistry and Biochemistry, Harper Cancer Research Institute, University of Notre Dame, Notre Dame, IN 46617, USA; thilliar@nd.edu; Tel.: +1-574-631-2452

**Keywords:** ovarian cancer, mesothelin, CA125, tumor microenvironment

## Abstract

Ovarian cancer is the deadliest gynecological disease among U.S. women. Poor 5-year survival rates (<30%) are due to presentation of most women at diagnosis with advanced stage disease with widely disseminated intraperitoneal metastasis. However, when diagnosed before metastatic propagation the overall 5-year survival rate is >90%. Metastasizing tumor cells grow rapidly and aggressively attach to the mesothelium of all organs within the peritoneal cavity, including the parietal peritoneum and the omentum, producing secondary lesions. In this review, the involvement of mesothelin (MSLN) in the tumor microenvironment is discussed. MSLN, a 40kDa glycoprotein that is overexpressed in many cancers including ovarian and mesotheliomas is suggested to play a role in cell survival, proliferation, tumor progression, and adherence. However, the biological function of MSLN is not fully understood as MSLN knockout mice do not present with an abnormal phenotype. Conversely, MSLN has been shown to bind to the ovarian cancer antigen, CA-125, and thought to play a role in the peritoneal diffusion of ovarian tumor cells. Although the cancer-specific expression of MSLN makes it a potential therapeutic target, more studies are needed to validate the role of MSLN in tumor metastasis.

## 1. Introduction

Ovarian cancer is the fifth leading cause of cancer death in U.S. women, making it the most lethal gynecological malignancy. The American Cancer Society estimates that about 22,240 new cases of ovarian cancer will be diagnosed in the United States in 2018, of which 14,070 (>60%) women will die of the disease [1]. The overall 5-year survival rate of women diagnosed with ovarian cancer is 47% and for women diagnosed with advanced stage disease, presenting with intraperitoneal metastasis, the 5-year survival rate is only 29% [1,2]. Ovarian cancer is a heterogeneous disease composed of seven histological subtypes: high-grade serous, low-grade serous, mucinous, endometrioid, clear cell, carcinosarcoma, and Brenner tumors [3]. Approximately 90% of ovarian cancers are classified as malignant epithelial ovarian carcinomas (EOCs), of which high-grade serous carcinomas (HGSC) account for 70% of tumor types [4,5,6,7]. Early signs or symptoms of ovarian cancer are often subtle and nonspecific which are frequently ignored or treated with medicine to relieve discomfort. In 50–80% of high-grade serous carcinomas, the most frequent genetic change is a p53 mutation found in tumors of all stages [8,9,10]. Mutations in BRCA1 and BRCA2, tumor suppressor genes, are found in about 50% and 70% of ovarian cancer patients with a family history of ovarian cancer, but 95% of ovarian cancer cases are sporadic [11,12,13].

**Figure 1:**
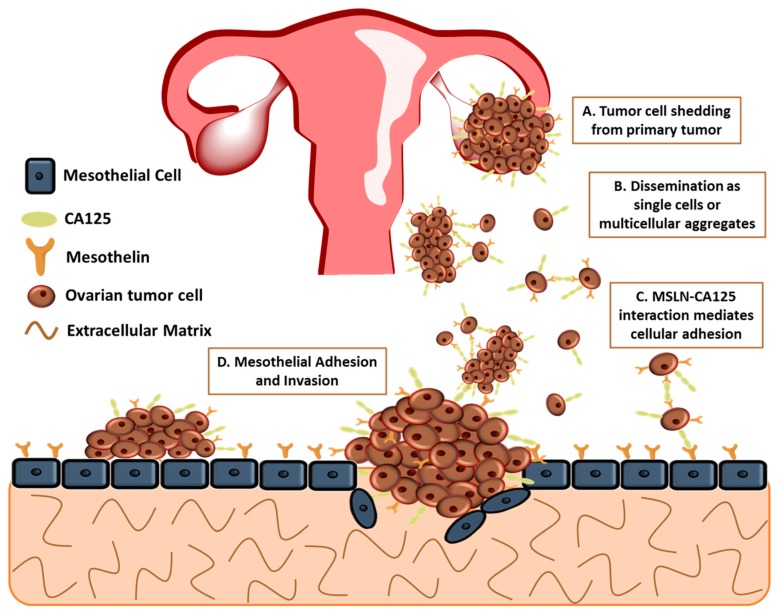
Model for peritoneal metastasis of ovarian tumors. Ovarian cancer metastasis is unique as tumor cells shed from the primary tumor and spread throughout the peritoneal cavity. MSLN:CA125 interaction mediates heterotypic and homotypic cellular adhesion.

The major cause of death is due to therapy-resistant metastasis from the primary tumor to the peritoneum [14,15,16,17,18]. The lack of successful eradication of the disease can be owing to the various complex overlapping signaling networks, together with the peritoneal tumor microenvironment composed of mesothelial cells, the submesothelial matrix, and adipose. Unlike other cancers, ovarian cancer uniquely metastasizes by the detachment of tumor cells, either single or multicellular aggregates, from the primary ovarian/fallopian tube tumor instead of the classically studied pattern of hematogenous metastasis (Figure 1A,B) [15,16,18,19]. Recent studies have challenged this mode of metastasis, suggesting that hematogenous spread of ovarian cancer may play a larger role in ovarian cancer cell metastasis; however, for the purpose of this review, ovarian cancer metastasis will be discussed as direct shedding of tumor cells [20,21]. This distinctive process bypasses several steps of intra- and extravasation before metastasis to other organs [19]. These detached cells undergo epithelial to mesenchymal transition before detaching, resulting in the loss of E-cadherin, a glycoprotein located at cellular junctions, and an invasive phenotype [22]. The metastatic cells disseminate throughout the peritoneal cavity, facilitated by natural fluid flow and preferentially attach to the mesothelium that covers all the organs in the peritoneal cavity including the omentum, abdominal peritoneum and the contralateral ovary (Figure 1C,D) [14,23,24]. Proliferation of disseminated tumor cells on the omentum eventually results in the obstruction of the bowel and stomach [25,26]. It is unknown if the primary tumor prepares secondary metastatic sites, including the omentum and peritoneum, for colonization, a process that has been implicated in other cancers [19]. 

Currently, there are no simple screening tests available to detect ovarian cancer. However, available diagnostic testing includes pelvic examinations, transvaginal ultrasonography and serum measurements of cancer antigen-125 (CA125) [27]. Identification of additional screening strategies to accurately diagnose patients in early stages are of great need. Moreover, mesothelin, a glycoprotein expressed in mesothelial cells and overexpressed in EOCs, could be useful as both a screening biomarker as well as a therapeutic target [28]. Understanding the interaction of the tumor and mesothelium and regulating the molecules that modify the metastatic tumor microenvironment is of great importance for the development of future therapeutics.

## 2. CA125

CA125, a repeating peptide epitope of the mucin 16 (MUC16), is a large membrane-bound cell surface mucin, discovered in 1981 by a monoclonal antibody OC125 developed from mice immunized with human ovarian cancer cells [29]. CA125 is a heavily glycosylated type I transmembrane protein belonging to the family of tethered mucins containing both *O*-linked and *N*-linked oligosaccharides [30]. CA125 is overexpressed in many tumors of epithelial origin suggesting that it plays an important role in tumorigenesis [30,31]. CA125 is commonly used as a biomarker to monitor ovarian cancer disease progression and relapse as it is highly expressed in ovarian carcinomas yet minimally expressed in normal ovarian tissues [32,33,34]. CA125/MUC16 has been shown to inhibit cytolytic responses of human natural killer cells in ovarian cancer, therefore acting as a suppressor of the immune response directed against the ovarian tumors [35,36]. CA125 has been shown to promote cancer cell proliferation [37]. Although the role of CA125 is mainly studied in ovarian cancer, recent studies have shown that CA125 is also highly expressed in other cancers including peritoneal mesotheliomas, pancreatic, and colorectal cancer, implicating a mesothelial cell interaction [38,39,40].

## 3. Mesothelial Cells

All organs of the abdominal cavity are covered by the mesothelium, a monolayer of mesothelial cells covering a basement membrane composed of fibronectin, collagen I and IV and laminin [41,42]. Mesothelial cells are flattened squamous-like cells derived from the mesoderm and possess both epithelial and mesenchymal characteristics [43,44]. Mesothelial cells have well-developed cell–cell junction complexes, including tight junctions, that are critical for cell surface polarity and the formation and maintenance of a semi-permeable diffusion barrier. The mesothelium functions to provide a protective barrier as well as a frictionless interface for the free movement of organs and tissues [45]. The mesothelium also plays an important role in contributing to the homeostasis of the peritoneal cavity, fluid and cell transport, tissue repair, initiation and resolution of inflammation and possibly tumor dissemination [46,47]. In the tumor microenvironment, mesothelial cells are preconditioned by the cancer cell secretome to induce the expression of multiple pro-inflammatory factors [48]. Mesothelial cells are implicated in both epithelial-to-mesenchymal transition (EMT) and mesothelial-to-mesenchymal transition (MMT), an EMT-like process [49,50,51]. EMT is the biological process by which epithelial cells lose cell–cell adhesion and gain migratory properties and MMT is a biologic process in which mesothelial cells of the peritoneal cavity acquire a fibroblast-like phenotype, with increased migratory capabilities [50,52]. Mesothelial cells, expressing mesothelin, line the peritoneal wall and all the organs of the peritoneal cavity that is susceptible to ovarian cancer metastasis.

## 4. Mesothelin

**Figure 2:**
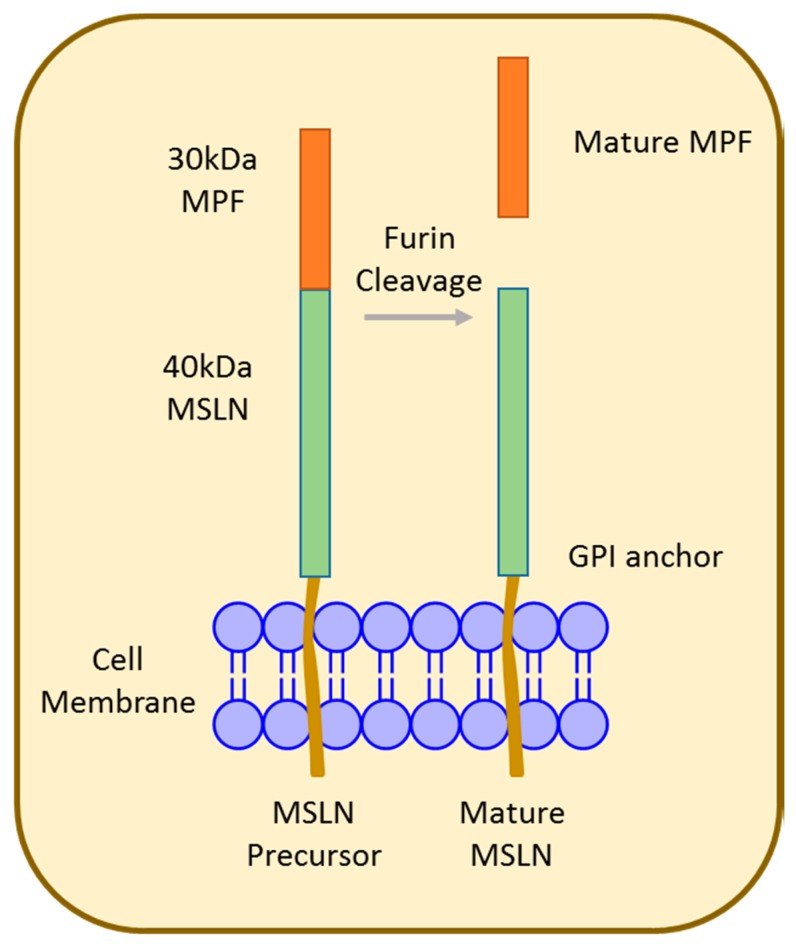
Structure of mesothelin (MSLN). The 70 kDa MSLN precursor protein is proteolytically cleaved to release the 30 kDa N-terminal megakaryocyte potentiating factor (MPF) and is displayed as mature MSLN on the cell surface.

Mesothelin (MSLN), first identified in 1992 [53], is synthesized as a 70 kDa precursor that is proteolytically cleaved at Arg295, resulting in an approximately 30 kDa fragment called megakaryocyte potentiating factor (MPF) and the 40 kDa MSLN membrane-bound fragment (Figure 2) [54,55]. Both MSLN and MPF are biologically active; however, the exact function remains unknown [56]. MSLN is a glycosylphosphatidylinositol (GPI)-anchored membrane glycoprotein that is physiologically expressed at the cell surface of mesothelial cells lining the pleura, pericardium, and peritoneum [57,58]. Composed of 16 exons spanning 7733 bp, the human MSLN gene occupies approximately 8 kb located at chromosome 16 p 13.3. Alternative splicing results in the predominant variant 1 encoded by MSLN1, variant 2 (24 bp insert), and variant 3 (82 bp insert) [55,57,59,60].

Although many prediction programs have attempted to predict the three-dimensional structure of the MSLN precursor and mature MSLN, the structure still remains unknown [61]. MSLN1 was found by Hellstrom et al. to be primarily expressed at the cell surface and was also released into body fluids of patients of several tumor types. Soluble MSLN results from a cleavage of variants 1 at the C-terminal domain [60]. An 18-bp enhancer sequence, CanScript, located −65 to −46 bp 5′ of one of three transcriptional start sites in the promoter region of the *MSLN* gene, was identified in cancer cell lines with aberrant overexpression of MSLN. The CanScript sequence enhancer consists of two functionally putative binding motifs: the conventional MCAT element and a SP1-like element [62]. All eight nucleotides in the MCAT element were shown to be essential for its function; conversely, the SP1-like element was shown to have two mutations suggesting, that the cancer-specific expression of MSLN is thought to occur through the binding of an unknown transcription factor. Transcription factors such as KLF6 and YAP1 have been investigated but binding of these factors are not adequate for MSLN overexpression in certain cancer types [63]. Nonetheless, the essential transcriptional factor that regulates the MSLN overexpression in human cancers has not been identified.

MSLN is normally expressed in mesothelial cells in trace amounts. In contrast, MSLN is highly expressed in human cancers including 70% of ovarian cancers [54,64,65,66], mesotheliomas [54], and pancreatic adenocarcinoma [67,68] and therefore identified as a tumor-associated marker. The biological function of MSLN is not fully understood as MSLN knockout mice do not present with an abnormal phenotype, suggesting that MSLN is a non-essential protein [58]. Furthermore, MSLN is reported to play a role in cell adhesion [69], tumor progression [65,70,71,72,73], and chemoresistance [73,74,75,76]. Specifically, MSLN has been shown to have oncogenic properties by increasing ovarian cancer invasion by inducing MMP-7 through MAPK/ERK and JNK pathways and by inducing drug resistance through PI3K/AKT and MAPK/ERK signaling pathways [65,74]. Albeit, mechanisms that regulate MSLN cell-surface expression are not well understood.

## 5. MSLN and CA125

CA125, the ovarian cancer antigen/biomarker, has been identified as a MSLN ligand and could potentially mediate cell adhesion [69]. Rump et al. demonstrated MSLN–CA125 interaction mediates heterotypic cellular adhesion (Figure 1C) of the human ovarian cancer cell line, OVCAR3, expressing CA125 to a MSLN expressing endothelial-like cell line [69]. Additionally, Gubbels et al. established that MSLN binds to CA125 in a specific and N-linked glycan-dependent manner, thus CA125-expressing ovarian tumor cells could bind specifically to the mesothelin-expressing peritoneal lining (Figure 1D) [77]. The *N*-linked oligosaccharides of CA125 are necessary for the binding to MSLN with MSLN having a strong affinity to CA125 with an apparent dissociation constant (K_d_) of 5 nM [77,78,79]. Consequently, MSLN:CA125-dependent cell attachment may play an important role in the peritoneal implantation of ovarian tumor cells [54,80]. The MSLN:CA125 role in cell attachment is supported by work from Bruney et al., demonstrating the overexpression of membrane type 1 matrix metalloproteinase (MT1-MMP) in human ovarian cancer cells (OVCA433-MT)-decreased cell surface expression of CA125/MUC16, subsequently increasing CA125/MUC16 ectodomain shedding, resulting in the release of CA125 from the cell surface. Additionally, there was decreased adhesion of OVCA433-MT to human mesothelial cells (LP9) and to intact peritoneal explants, suggesting the importance of MSLN:CA125 initial adhesion of [81]. After initial attachment of ovarian cancer cells to the peritoneal mesothelium, the co-overexpression of both MSLN and CA125 can lead to recruitment of other ovarian cancer cells being sloughed off from the primary site (Figure 1B,C) [82]. Therefore, the tumor load at secondary sites could be a combination of excessive proliferation and adhesion of circulating single or multicellular aggregates in peritoneal ascites fluid [77,83]. Conversely, the exact function of MSLN in tumor progression remains unclear [84]; however, understanding the importance of CA125:mesothelin binding may lead to novel therapies to control ovarian peritoneal metastasis.

## 6. Targeting MSLN

Clarifying the function of MSLN will enhance its clinical application in ovarian cancer, including early detection, chemo-response, prognosis and therapeutic targeting. Several features of MSLN make it a useful candidate for cancer therapy, including that it is well-internalized, enabling it to be a good target for immunotoxins [85]. Additionally, MSLN is actively shed from the cell surface generating a pool of antigens in ascites or blood circulation allowing for the quantification of circulating serum MSLN levels potentially used for diagnosis of ovarian cancer patients [28,86,87,88]. The use of MSLN as a plasma biomarker has been investigated by several groups using blood ELISA tests and demonstrated that serum MSLN levels decrease after surgical therapy and, therefore, may be useful in monitoring treatment response in MSLN expression cancers [86,89]. Pools of antigens, from shed MSLN, in the tumor interstitial space will unavoidably interact with a targeting agent during tumor dissemination [60,85,87]. The first identified sheddase, TNF-α converting enzyme (TACE) was shown to mediate MSLN shedding. TACE is a transmembrane glycoprotein, known for its role in releasing EGFR ligands from the cell surface, therefore regulating the activation of the EGFR pathway [60,90]. Tumor targeting is a complex process and, furthermore, modulation of MSLN shedding could have an influence on drug kinetics in both circulation and tumor tissue. However, shedding is not the only way MSLN could be modulated. The expression levels of MSLN could potentially be regulated similarly to other antigens by trogocytosis [91] or antigen masking [92]; however, the role of these antigens remains to be elucidated. Furthermore, MSLN is expressed in dispensable mesothelial cells so the risk of non-specific toxicity is decreased.

### 6.1. Molecular Imaging for the Detection of MSLN

Mesothelin has recently been investigated as a target for molecular imaging probes. These probes are designed to guide antibody-based treatments that can be used to assess tumor uptake, response to treatment and the distribution in primary tumors and secondary sites. Prantner et al. identified and characterized an antimesothelin nanobody (NbG3a) used for in vitro diagnostic applications [93]. Further studies from the same group established the potential use of NbG3a for a novel molecular imaging probe with promising results for human imaging and therapeutic applications [94]. Terwisscha van Scheltinga et al. investigated the use of an antibody–drug conjugate (anti-mesothelin antibody-monomethyl auristatin E) coupled to molecular imaging with ^89^Zr immuno-positive emission tomography (PET). Using this technique, quantitative immuno-PET measurement of relative antibody uptake was determined to correlate with tumor growth inhibition [95]. Furthermore, non-antibody protein scaffolds have successfully been engineered to bind to mesothelin with high affinity [96]. Unlike antibodies that are large in size and have slow clearance from circulation, non-antibody protein scaffolds have demonstrated specific binding to identify tumors expressing the molecular target in murine models [97,98,99] and have demonstrated promising results in both preclinical and clinical evaluations [100]. The use of these techniques demonstrates the translational potential of MSLN.

### 6.2. Clinical Trials

There are many clinical trials testing MSLN-targeting agents using strategies such as antibody-based immunotoxins such as SS1P, consisting of an anti-MLSN Fv obtained from a phage display library of immunized mice with recombinant MSLN fused to a truncated form of the Pseudomonas Exotoxin PE38 that mediates cell death. The mechanism of action of an immunotoxin is threefold. First, the immunotoxin binds to cell-bound MSLN; second, this complex is internalized by endocytosis, undergoes retrograde transport to the endoplasmic reticulum and the PE portion is translocated to the cytosol; and third, the PE catalyzes ADP-ribosylation of the elongation factor-2, halting protein synthesis and activating apoptosis [85,101]. There have been two Phase I clinical trials with different modes of administration using either continuous infusion or as bolus intravenous infusions in mesotheliomas, ovarian, and pancreatic cancers. Continuous infusion was well tolerated and showed modest clinical activity; however, there was advantage seen over bolus dosing [102,103]. Additionally, there is a high affinity chimeric antibody, amatuximab (MORAb-009), with high affinity and specificity for mesothelin that is under investigation in clinical trials. Amatuximab works by inducing antibody-dependent cellular cytoxicity [104]. It was observed that upon treatment with amatuximab, patients had an increase in CA125 levels suggesting that amatuximab interferes with the MSLN:CA125 interaction [105]. A tumor vaccine CRS-207 utilizing a live attenuated strain of bacterium *Listeria monocytogenes* (*Lm*) expressing human MSLN has shown good tolerance and MSLN-specific T-cell response in a phase I study of safety clinical trial. This phase I study not only demonstrated that vaccines are safe and tolerable but also showed that a tumor antigen-modified *Lm* can induce tumor antigen-specific T-cell responses in patients with advanced cancer, suggesting that further evaluation of *Lm* vaccine as a candidate biomarker of improved clinical outcomes is needed [106]. A two-part phase I/II trial is underway using combination therapy with CRS-207, epacadostat, and pembrolizumab (keytruda) in patients with platinum-resistant ovarian, fallopian tube, and peritoneal cancers using different combinations of the three treatments (ClinicalTrials.gov Identifier NCT02575807). Antibody–drug conjugates is another strategy used to target MSLN. An ongoing phase I clinical trial with anetumab ravtansine (BAY94-9343) to determine the safety and maximum tolerated dose for patients with advanced solid tumors including ovarian carcinoma and mesothelioma opened in 2011 (ClinicalTrials.gov Identifier NCT01439152). Anetumab ravtansine consists of the fully human anti-MSLN IgG1 linked to a potent tubulin-binding drug, DM4. In preclinical trials, anetumab ravtansine inhibited both subcutaneous and orthotopic tumor growth in xenograft models of ovarian, pancreatic, and mesothelioma cancers [107]. In patients with recurrent MSLN-expressing platinum-resistant recurrent ovarian, fallopian tube or primary peritoneal cancer, a phase Ib clinical trial to determine the maximum tolerated dose of anetumab ravtansine that could be safely combined with pegylated liposomal doxorubicin is underway (ClinicalTrials.gov Identifier NCT02751918). Several ongoing clinical trials are utilizing anti-MSLN CAR-modified T cells as MSLN targeting agent. The T cells are obtained by apheresis and introduced to a temporary gene which will cause them to make a new type of antibody that will attach to MSLN. Once attached, the cells will become activated and stimulate the host immune system to attack the MSLN-expressing cells [108]. The above clinical trials have confirmed that targeting MSLN could be beneficial in improving existing therapeutic options for patients diagnosed with a MSLN-expressing cancer, including ovarian cancer.

## 7. Conclusions

Ovarian cancer is the deadliest gynecological malignancy among U.S. women and is often diagnosed at a late stage when the disease has metastasized into the peritoneal cavity. Mesothelin, a glycoprotein normally expressed in mesothelial cells, is highly expressed in several cancers including ovarian, pancreatic, and mesotheliomas. It has been shown that MSLN binds to the ovarian cancer biomarker CA125 and this interaction plays a role in the peritoneal metastasis of ovarian cancer. The differential expression of mesothelin in normal and cancer tissues makes it a promising candidate for targeted therapeutics. Several candidate immunotherapies targeting MSLN are in ongoing clinical trials. New strategies to disrupt the MSLN:CA125 interaction are emerging. Although MSLN is implicated in many cancers, the role of MSLN is still poorly understood warranting further investigation and clinical trial studies. Future advances in ovarian cancer therapy depend on novel treatment mechanisms in combination with current chemotherapeutic approaches that will result in cytotoxicity, inhibition of metastasis and angiogenesis, and increasing the immunological detection of tumors. Further mechanistic studies on MSLN are needed to validate the potential role of MSLN in tumor metastasis that possibly will provide insight for effective MSLN-targeting therapies for several cancers.
